# Ready to detect a reversal of time's arrow: a psychophysical study using short video clips in daily scenes

**DOI:** 10.1098/rsos.230036

**Published:** 2023-04-19

**Authors:** Nao Hanyu, Kei Watanabe, Shigeru Kitazawa

**Affiliations:** ^1^ Dynamic Brain Network Laboratory, Graduate School of Frontier Biosciences, and; ^2^ Department of Brain Physiology, Graduate School of Medicine, Osaka University, Osaka 565-0871, Japan; ^3^ Center for Information and Neural Networks (CiNet), National Institute of Information and Communications Technology, Osaka 565-0871, Japan

**Keywords:** time's arrow, time perception, natural video stimuli, diffusion model, Rayleigh distribution, reaction time

## Abstract

It is generally believed that time flows in one direction and that a reversal of time's arrow would render the external world non-sensical. We evaluated our ability to tell the direction of time's arrow in a wide range of dynamic scenes in our daily life by presenting 360 video clips in the correct or incorrect direction. Participants, who judged the direction in a speeded manner, erred in 39% of trials when a video was played in reverse, but in only 9% when it was played normally. Due to the bias favouring the ‘forward’ judgement, the reaction was generally faster for the forward response. However, the reaction became paradoxically faster and more synchronous for the detection of reversal in some critical occasions such as forward motion, free fall, diffusion, division and addition of materials by hand. Another experiment with a fraction of the video clips revealed that reversal replay of these videos provided instantaneous evidence strong enough to overtake the forward judgement bias. We suggest that our brain is equipped with a system that predicts how the external organisms behave or move in these critical occasions and that the prediction error of the system contributes to the fast ‘reversal’ detection.

## 1. Introduction

An English proverb says, ‘it is no use crying over spilt milk’. We substitute milk with water in Japanese, saying ‘spilt water does not return to the vase’. The Japanese proverb did not originate in Japan but was borrowed from a historical document in ancient China. The existence of these proverbs tells us that it is universal and long-lasting knowledge, or belief, that time flows in one direction and not in reverse. In the twentieth century, Sir Arthur Eddington, a British astrophysicist who introduced the phrase ‘time's arrow’, noted that ‘our reasoning faculty tells us that a reversal of the arrow would render the external world nonsensical’ [1]. Indeed, we would feel it non-sensical if water on the ground would jump up into the vase. However, it is not clear how and to what extent our reasoning faculty, the brain, could tell the correct direction of the arrow from the incorrect.

It is the proper direction of time's arrow that underlies our judgement of the order of any events in time. It is generally accepted that we are able to judge the order of successive stimuli when they are separated by 20–30 ms [2,3], showing that we are sensitive to the direction of time's arrow up to this fine time scale in general. However, the judgement could be modified by attention [4,5] or prior experiences [6–8] in that two simultaneous stimuli could be judged as having occurred sequentially [4,5] or two stimuli separated by 20–80 ms were judged as simultaneous [6–8]. These studies have shown that the flow of subjective time is flexible as compared to that measured by the clock. Furthermore, subjective temporal order could even be reversed: two successive touches to the hand could be reversed by just crossing the hands even when the separation of two stimuli was as large as 100–500 ms [9–11]. These studies clearly show that the flow of subjective time or even the direction of time's arrow is far from being stable. It raises a possibility that our ability to judge the direction of time's arrow is quite limited on some occasions even though we believe that ‘a reversal of the arrow would render the external world nonsensical’.

In the present study, we aimed to evaluate our ability to tell the direction of time's arrow in a wide variety of natural scenes. For this purpose, we used 360 short video clips, 3 s in duration, in the ‘Moments in Time Dataset’ [12], which involve ‘people, animals, objects or natural phenomena, that capture the gist of a dynamic scene’ in our daily life. In the first experiment, we asked participants to judge whether the 3 s video clip was played in the correct direction or in the reverse in a rapid manner. We show that the ability to identify reversal was not perfect (erred in 39% of trials) but depended strongly on a few critical cues, such as free fall of objects, forward motion of people, animals and vehicles, diffusion of materials, and division or addition of materials by hand. However, these cues, or evidence for judgement, were not evenly distributed over time. This results in marked contrast with artificial stationary visual stimuli, such as random dot motion stimuli, which are often used to explore neural mechanisms of decision making [13,14]. In the second experiment, we evaluated dynamic changes in the strength of evidence in 3 s by presenting a fraction of the video (300 ms in duration) and improved the temporal resolution up to 100 ms. Based on the results of the two experiments, we finally developed a quantitative model of judging the direction of time's arrow using time-dependent hazard rate functions [15].

## 2. Material and methods

### 2.1. Participants

Thirty-seven healthy adults (aged 20–27 years), 10 for Experiment 1 and 27 for Experiment 2, participated. All participants were right-handed and had normal or corrected-to-normal visual acuity. All participants provided written informed consent. The study was approved by the ethics committee of the Graduate School of Frontier Biosciences of Osaka University, Japan.

### 2.2. Stimuli and experimental procedures

**Figure 1.**
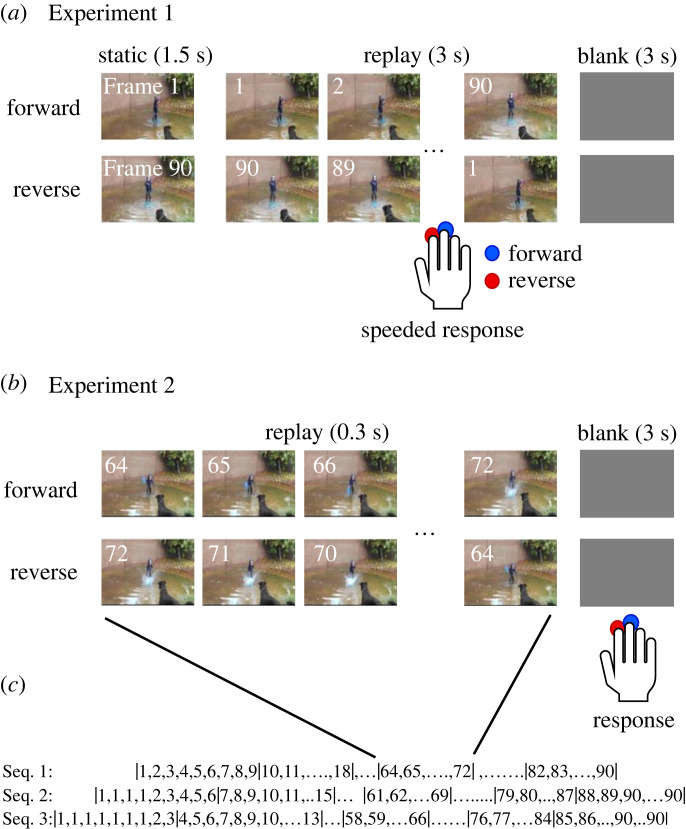
Experimental procedures. (*a*) Experiment 1. Each video clip was presented in the forward or reverse direction for 3 s after the first frame was presented for 1.5 s. Participants made a rapid response as soon as they judged the direction by pressing a button. (*b*) Experiment 2. Only a fraction of video that consisted of nine frames was presented in either direction for 300 ms. Participants made a response during the blank period after the presentation (3 s). (*c*) Three sequences of 300 ms fractions in Experiment 2. The first fraction was {Frame 1, 2, … ,9} for sequence 1, {Frame 1, 1, 1, 1, 2, … ,6} for sequence 2 and {Frame 1, 1, 1, 1, 1, 1, 2, 3} for sequence 3. Multiple 1's mean that Frame 1 was used as a filler of empty frames. A fraction from sequence 1, {64, 65, … , 72}, is shown as an example in (*b*).

In Experiment 1, we used 360 video clips (3 s long, 29.97 fps, 90 frames) that were selected from the ‘Moments In Time dataset’ [12]. The database consists of 339 different classes, each with one action label, such as ‘picking’, ‘running’, ‘closing’ and ‘playing’. We chose one from each class (*n* = 339) and 21 more (one from each of 21 randomly chosen classes) to cover a wide variety of dynamic scenes of daily life. In choosing one from each class, our choice was essentially random, except that we excluded videos with panning, zooming or scene changes. Each video clip was presented on a tangent liquid crystal display (Dell P190Sb, refresh rate = 60 Hz, 1280 × 1024 pixels) in either forward (from frame No. 1 to 90) or reverse (from No. 90 to 1) direction after presenting the first frame (No. 1 or No. 90) for 1.5 s (figure 1*a*). Participants, seated on a chair with their head resting on a chin rest, viewed each video (600 × 340 pixels, 25.1° × 14.4° in the viewing angle) on the display placed at a viewing distance of 40 cm and judged whether each video clip was played in the forward or reverse direction. Participants were required to respond as soon as they reached a judgement by pressing one of two buttons that were assigned to the index finger (forward) and the middle finger (reverse) of the right hand (figure 1*a*). They were allowed not to respond when they did not reach a judgement, but they responded in 98.8% of trials within 5 s of the movie onset. Each video clip was played twice, once in the forward and once in the reverse direction, in two different sessions that were performed on two different days. Participants performed 360 trials per day, in which half (*n* = 180) were played in forward and the others were played in reverse. Permutations were randomly determined for each participant.

In Experiment 2, we chose 120 video clips from the 360 clips used in Experiment 1. Sixty-six of the 120 video clips were video clips with perfect judgement across the 10 participants in Experiment 1 (evident videos). The other 54 clips were chosen from 63 video clips in which the correct judgement rate was equal to or smaller than 0.5 (ambiguous videos). Only a fraction (300 ms, 30 fps, 9 frames) of the video clips were presented in each trial, and participants were required to judge the direction and respond during a period of 2 s after each presentation (figure 1*b*). Each video clip was divided in three different ways into 10 or 11 fractions: type (1) {Frame 1–9}, {10–18}, … , {82–90} (10 fractions), type (2) {1, 1, 1, 1–6}, {7–15}, … , {79–87}, {88–90, 90, 90, 90, 90, 90, 90} (11 fractions), and type (3) {1, 1, 1, 1, 1, 1, 1–3}, {4–12}, … ., {76–84}, {85–90, 90, 90, 90} (figure 1*c*). Here, {1, 1, 1, 1–6} means that there were three Frame 1's, three fillers, before Frames 1, 2, 3, 4, 5 and 6. Frame 1 was used as fillers of three empty frames because Frames 1–6 cannot fill all nine frames. Likewise, Frame 90 was used as fillers of the right-most empty frames when necessary. The borders of divisions were different by three frames (100 ms) to improve the overall temporal resolution from 300 to 100 ms. For each of the 120 video clips, nine participants were assigned, and three were assigned to each of the three types of divisions (3 × 3 = 9). To reduce the number of trials for each participant, we recruited 27 participants and showed a part of the 120 video clips, 40 (one-third of 120) on average, to each participant. To be more precise, nine participants (Group 1) were assigned to 29 video clips (14 evident and 15 ambiguous), nine (Group 2) to 45 (26 evident and 19 ambiguous) and the other nine (Group 3) to 46 (26 evident and 20 ambiguous) video clips. Each participant participated in two sessions over two consecutive days and made judgements in 380 to 506 trials per day. The number varied according to the type of division and video clips assigned to each group of participants. The order of presentations was randomized for each participant. The total number of trials was 23 040 (= 120 video clips × (10 + 11 + 11) fractions×2 directions × 3 participants). In some stimuli, there was actually no movement at all, in which case participants were allowed to press both buttons to express that they did not see any movement. Participants responded by pressing one of the two buttons in 95.6% (*n* = 22 018) of the trials (*n* = 23 040).

Tasks were controlled using MATLAB (version 2015b, MathWorks) and Psychtoolbox (version 3.0.15, 1997), which were run on a Windows PC (Dell Precision 3430, Windows 10 pro 64 bit 21H1). The timing of the button press was acquired at a sampling rate of 1000 Hz using a USB input/output board (NI USB-6229, National Instruments) connected to the PC.

### 2.3. Data analysis

In Experiment 1, we calculated the probability of correct judgement, p(f|f) and p(r|r), for each of the 360 video clips by accumulating responses from 10 participants. Here, p(f|f) denotes the probability of the ‘forward’ response in response to ‘forward’ presentation. By applying signal detection theory [16] to p(f|f) and p(r|r), we calculated the level of separation (*d′*) and response bias (positive for the bias favouring the forward judgement) by using the formula as follows:

2.1 $$d^{\prime }=norminv(p(\;f|f))+norminv(p(r|r))$$

and

2.2 bias=(norminv( p( f|f))−norminv( p(r|r))/2,

where *norminv* denotes the normal inverse cumulative distribution function. We substituted 0.01 and 0.99 for 0 and 1, respectively, to prevent the values from diverging to infinity. These values were calculated for each video clip and for all 360 video clips as a whole.

Then, we defined evident videos with perfect judgement (p(f|f) = 1 & p(r|r) = 1, *d′* > 4) and ambiguous videos with an overall correct judgement rate of 0.5 or smaller (p(f|f) + p(r|r) ≤ 1, *d′* ≤ 0). The reaction time (RT), measured from video onset, was accumulated across the participants for each of 2-by-2 groups of trials: (evident or ambiguous) × (forward or reverse responses). Distributions of the RT were fitted by the Rayleigh distribution with a fixed delay for each group to see if there were any significant differences in the speed of judgement. Two parameters (delay and a peak time of the Rayleigh distribution, 1/b s) were estimated using maximum-likelihood estimation. We also estimated 95% confidence intervals of the peak RT. The lack of overlap between two 95% confidence intervals was used as a good indicator of separation because the two groups could be separated with specificity greater than 0.975 and sensitivity (statistical power) greater than 0.975. We additionally fitted each distribution of RT using a diffusion model with a single threshold (time step = 1 ms, step size = + 1 or −1, starting position = 0) and a normal distribution [17]. Two parameters (p: probability of choosing +1 and threshold for the diffusion model and the mean and the standard deviation for the normal distribution) were estimated by using maximum-likelihood estimation. The three models (Rayleigh with delay, diffusion and normal distribution) with the same degrees of freedom (d.f. = 2) were compared using the negative log-likelihood (the smaller the better). We chose the Rayleigh distribution model after comparing the model with a diffusion model with a single threshold and a normal distribution model because the Rayleigh model performed best in fitting three of the four distributions of the RT in terms of log-likelihood (electronic supplementary material, figure S1). We also compared the standard deviation of the response across the four groups. Wilcoxon rank sum tests were used to compare six pairs of medians with Bonferroni corrections. The effect size was measured using the area under the curve (AUC).

In Experiment 2, we quantified the strength of evidence that each fraction of video had by combining responses from nine participants for each of the 120 video clips for each direction of presentation. For this purpose, we assigned a value of +1 to nine frames of a fraction if a participant judged the fraction as ‘forward’, and a value of −1 if a participant judged otherwise. By summing the values across nine participants and dividing the sum by 9, we obtained an ‘evidence score’ that took a value from −1 (all participants judged as reverse) to +1 (all judged as forward).

We then developed a quantitative model to predict the response probability density (1/s) observed in Experiment 1 based on the evidence score obtained in Experiment 2. To calculate the response probability density, we introduced a hazard rate function (*h_f_* for the forward and *h_r_* for the reverse response) whose slope is modified by the evidence score as follows:

2.3 $$h_{f}(t+\mathrm{delay})=t\sqrt{{\left\{k_{f}\;\max(es(t)-c,0)\right\}}^{2}+{\left(1/b_{f}\right)}^{2}}$$

and

2.4 $$h_{r}(t+\mathrm{delay})=t\sqrt{{\left\{k_{r}\min(es(t)-c,0)\right\}}^{2}+{\left(1/b_{r}\right)}^{2}}.$$

Here, *c* denotes a neutral bias of the evidence score at which the hazard rate functions simply correlate with time and yield the Rayleigh distributions with a peak at *b_f_* and *b_r_*. The response probability density per unit time (pdf_f and pdf_r) was calculated using the following equations:

2.5 $$\tilde{b_{f}}(t)=1/\sqrt{{\left\{k_{f}\;\max(es(t)-c,0)\right\}}^{2}+{\left(1/b_{f}\right)}^{2}}$$

2.6 $$\tilde{b_{r}}(t)=1/\sqrt{{\left\{k_{r}\min(es(t)-c,0)\right\}}^{2}+{\left(1/b_{r}\right)}^{2}},$$

2.7 $$h(t)=h_{f}(t)+h_{r}(t),$$

2.8 $$pdf(t)=h(t)\;\exp\left\{-\int_{0}^{t}h(t)\;dt\right\},$$

2.9 $$B_{f}(t)=\tilde{b_{r}}{\left(t\right)}^{2}/\{\tilde{b_{f}}{\left(t\right)}^{2}+\tilde{b_{r}}{\left(t\right)}^{2}\},$$

2.10 $$B_{r}(t)=\tilde{b_{f}}{\left(t\right)}^{2}/\{\tilde{b_{f}}{\left(t\right)}^{2}+\tilde{b_{r}}{\left(t\right)}^{2}\},$$

2.11 $$pdf\_f(t)=B_{f}(t)pdf(t)$$

2.12 $$\mathrm{and}\;\;pdf\_r(t)=B_{r}(t)pdf(t).$$

We fitted the hazard rate model to the data of 120 video clips in Experiment 1 using maximum-likelihood estimation. To ensure the generalizability of our data fitting, we used the leave-one-out technique. Specifically, we performed 240 fits, one for each of the 120 video clips that were replayed in both forward and reverse directions, excluding the dataset in question from each fit. Data analyses were performed using in-house programs run in MATLAB (R2021b, MathWorks).

### 2.4. Estimation of *d′* from annotation of video clips

By observing the evident videos (*n* = 66) one by one, we noticed five categories of motion: (1) forward motion of people, animals, or vehicles, (2) free fall, or ballistic motion, under gravitational force, (3) centrifugal diffusion or explosion of small particles, (4) division of material by hand or a tool and (5) addition, or construction of material on others by hand. We also noticed that many ambiguous videos contained (6) reciprocating motion. Three experimenters rated the 360 videos as to whether there was each category of motion (1) or not (0). The six scores were averaged across raters (*x*_1_, *x*_2_, … .*x*_6_) and used to predict the *d′* of each of the 360 video clips. A linear model with the six parameters and a constant was fitted to the data as follows:

2.13 $$d^{\prime }∼1+x_{1}+x_{2}+x_{3}+x_{4}+x_{5}+x_{6}.$$

Six coefficients and their standard errors were estimated using the *fitlm* function in MATLAB. T-tests were used to test whether each of the estimated coefficients was significantly different from zero. The 95% confidence interval of each coefficient (1.96 times the standard error) was used to test whether the coefficient was significantly different from the others.

## 3. Results

**Figure 2.**
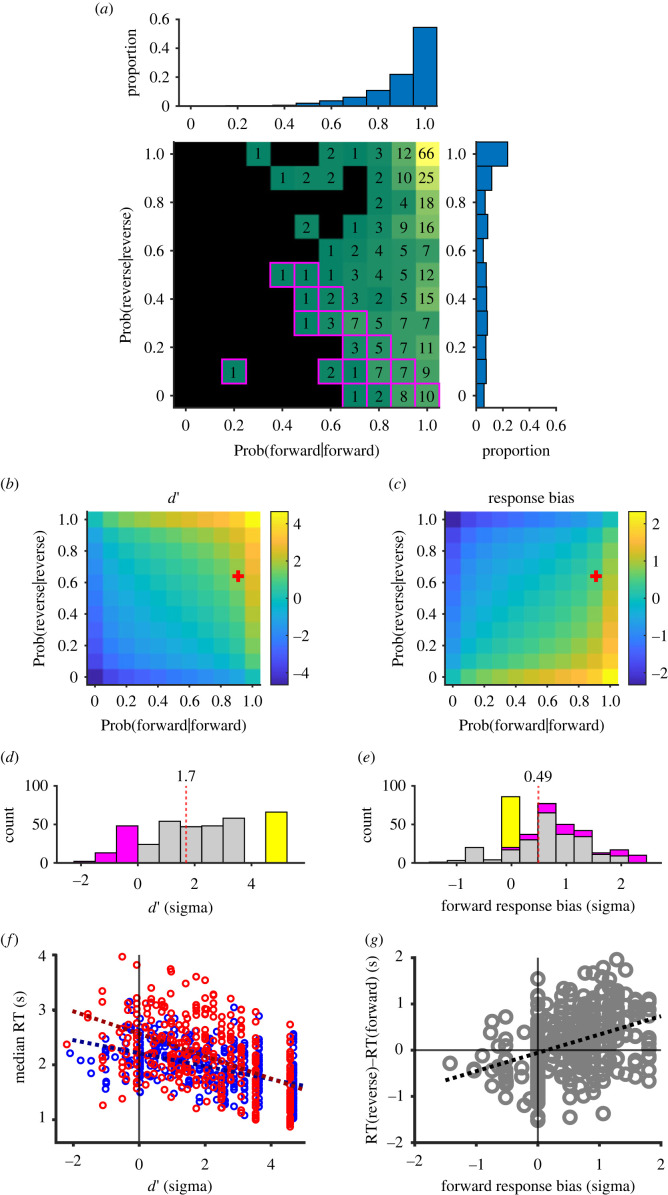
Responses to 360 video clips in Experiment 1. (*a*) Distributions of correct judgement probabilities, one for the forward replay (Prob(forward|forward), abscissa) and the other for the reverse replay (Prob(reverse|reverse), ordinate). The number of video clips with a combination of (Prob(forward|forward), Prob(reverse|reverse)) is shown in each tile. There were 66 video clips with perfect judgement, (1, 1), as shown in the top-right corner (evident videos). There were 63 video clips with a correct judgement rate less than or equal to 0.5 (demarcated by a magenta line, ambiguous videos). Histograms show the marginal distributions. (*b*,*d*) Distributions of *d′*. *D′* was maximized (greater than 4) with perfect judgement, (1, 1) and neutralized (0) with a correct judgement rate of 0.5. Using the *d′*, the evident videos could be defined as those with the maximum *d′* (yellow bar of the histogram (*d*)) and ambiguous videos as those with *d’* ≤ 0 (magenta). The overall *d′* calculated at (0.91, 0.64) was 1.7 (cross in (*b*), and vertical dotted line in (*d*)). (*c*,*e*) Distributions of response bias. Note that the majority of video clips yielded positive response bias favouring the forward judgement. The overall response bias at (0.91, 0.64) was 0.49 (red cross in (*c*) and vertical dotted line in (*e*)). (*f*) Median RT plotted against *d′* for each video clip. Each video was plotted twice, once for forward responses (blue circles) and once for reverse responses (red circles). Regression lines are superimposed for forward (blue dotted line) and reverse responses (red dotted line). (*g*) Difference in the median RT (reverse RT – forward RT) plotted against response bias. Each video is plotted once. Note that the majority of videos showed positive response bias favouring the forward response that was associated with the positive difference in RT, that is, faster forward responses than reverse responses. Note a positive slope of a regression line.

In Experiment 1, 10 participants were asked to judge whether each of 360 video clips was played in forward or in reverse in a speeded manner (figure 1*a*). The probability of correct judgement in the forward replay trials (p(f|f)) and in the reverse replay trials (p(r|r)) for each of the 360 video clips distributed rather divergently on the p(f|f) – p(r|r) plane (figure 2*a*). However, the marginal distribution of p(f|f) was strongly skewed toward the perfect judgement of 1 (0.9, 1 and 1 for the 25th, 50th and 75th percentiles), whereas p(r|r) was distributed widely from zero to one (0.3, 0.7 and 0.9). As a whole, participants erred in 39% of trials when a video was played in reverse (p(f|r) = 0.39; p(r|r) = 0.61), whereas they erred in only 9% when it was played normally (p((r|f) = 0.09, p(f|f) = 0.91), showing that there was clear bias favouring the forward judgement (forward bias). The forward bias, calculated by using signal detection theory, was 0.49 as a whole (cross in figure 2*c*, dotted line in figure 2*e*), and the majority of the video clips yielded forward bias when the bias was calculated for each video clip (figure 2*e*). *D′*, a separation of two hypothetical normal distributions representing the two judgements, was 1.7 as a whole (cross in figure 2*b*, and dotted line in figure 2*d*). It was remarkable that there were 66 videos that yielded the maximum *d′* (figure 2*d*, bar in yellow), where all 10 participants made perfect judgements about whether the clips were played forward or in reverse (yellow tile in figure 2*a*). We termed these 66 video clips with perfect judgements ‘evident’ video clips. We also defined ‘ambiguous’ video clips as those with the *d*′ equal to or less than 0 (figure 2*d*, magenta, tiles circumscribed with magenta in figure 2*a*).

When we plotted the median RT against the *d′*, it was clear that the RT decreased as the *d′* increased (figure 2*f*), as shown by regression lines with negative slopes, one for forward judgement (blue, slope = −0.12, *r* = 0.50, *p* < 0.0001) and another for reverse judgement (red, slope = −0.21, *r* = 0.53, *p* < 0.0001). These observations show that the RT became shorter as the evidence for the judgement increased. Interestingly, the intercept of the regression line for the forward judgement (blue dotted line, 95% c.i. = [2.0, 2.2] s) was significantly shorter than the other for the reverse judgement (red dotted line, c.i. = [2.4, 2.6] s). This result shows that participants generally made faster forward responses when there was no evidence for discrimination (*d′* = 0), which naturally follows from the forward response bias. The tendency was directly confirmed when we plotted the difference in the RT (RT of the reverse response – RT of the forward response) against the forward response bias (figure 2*g*). The RT difference (positive for shorter forward response) correlated with the forward response bias (slope = 0.40, *r* = 0.38, *p* < 0.0001) with an intercept that was not significantly different from zero (95% c.i. = [−0.14, 0.03] s). Taken together, participants showed response bias favouring the forward judgement and made faster forward responses in general.

**Figure 3.**
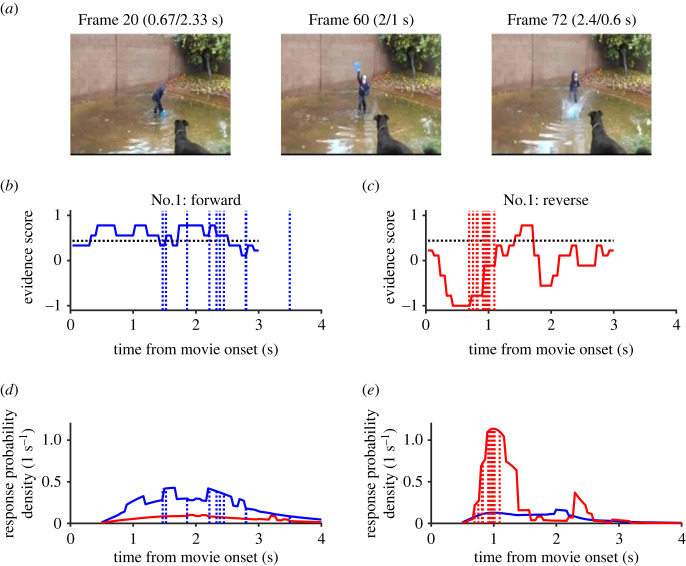
An evident video clip (No. 1). (*a*) Three critical frames at which a boy snatched a bucket (frame 20 at 0.67 s), wound it up to the top (frame 60 at 2 s) and slammed it down onto the surface of the water (frame 72 at 2.4 s) when the video was played in the normal direction. When it was played in reverse, frames 72, 60 and 20 were presented at 0.6, 1 and 2.33 s, respectively. Numbers in parentheses show the time of appearance in the forward and reverse replay trials. (*b,c*) Responses (Experiment 1, vertical broken lines) and the evidence score (Experiment 2, thick stair-like lines) when the video was played forward (*b*) and in reverse (*c*). Note synchronous responses at approximately 0.7–1.1 s and a sharp trough of the evidence score at approximately 0.5 s in (*c*). A horizontal dotted line shows the neutral bias of the evidence score (0.47) estimated by the hazard function model shown in equations (2.1) and (2.2). (*d*,*e*) Response probability density functions per second estimated using the hazard function model. Blue traces show the density function for the forward response (pdf_f), and red traces show the reverse response (pdf_r). Note in (*e*) that pdf_r has a sharp peak at approximately 1 s, and actual responses (vertical lines) occurred during the peak.

In one evident video clip (No. 1), a boy, standing in a puddle, snatched a bucket (frame 20, figure 3*a*), held it high (frame 60) and slammed it down on the surface of muddy water (frame 72), making a splash. During the forward replay, participants made correct judgements over a period of 2 s, from 1.5 s to 3.5 s (mean = 2.3 s, s.d. = 0.62 s), mostly during the latter half of the replay (blue dotted vertical lines, figure 3*b*). On the other hand, they made faster and more synchronous judgements of ‘reverse’ during a short period from 0.69 s to 1.1 s (mean = 0.90 s, s.d. = 0.12 s, red dotted lines, figure 3*c*). These synchronous responses appeared to have occurred in response to an evident cue, unnatural convergence of the splash, at approximately 0.6 s.

**Figure 4.**
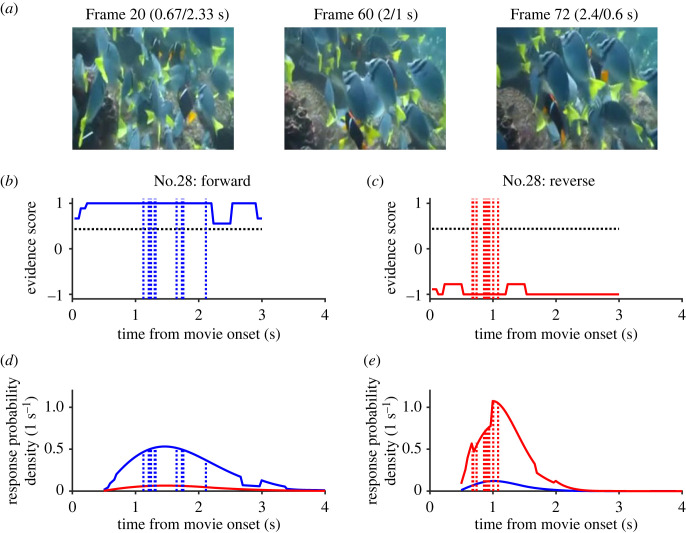
Another evident video clip (No. 28). (*a*) Three frames at the same timings as those in figure 3. Note that tropical fishes were continuously swimming forward through the video. (*b,c*) Responses and evidence scores. Note that the evidence scores were saturated to +1 (forward) and −1 (reverse) from the beginning, but reverse responses were faster and more synchronous (*c*) than forward responses (*b*). (*d*,*e*) Response probability density functions per second estimated using the hazard function model. Note the contrast of dull (*d*) and sharp (*e*) peaks predicted from the model, which matched well with actual forward and reverse responses.

In another evident video clip (No. 28), tropical fishes were swimming in the sea (figure 4*a*). Though their movements were continuous and obvious from the beginning of the video clip, participants made slower and more divergent responses during the forward replay (1.1–2.1 s) than during the reverse replay (0.7–1.1 s, figure 4*b,c*). It is worth noting that the reverse responses were faster and more synchronous than the forward responses though the forward response bias predicts otherwise.

**Figure 5.**
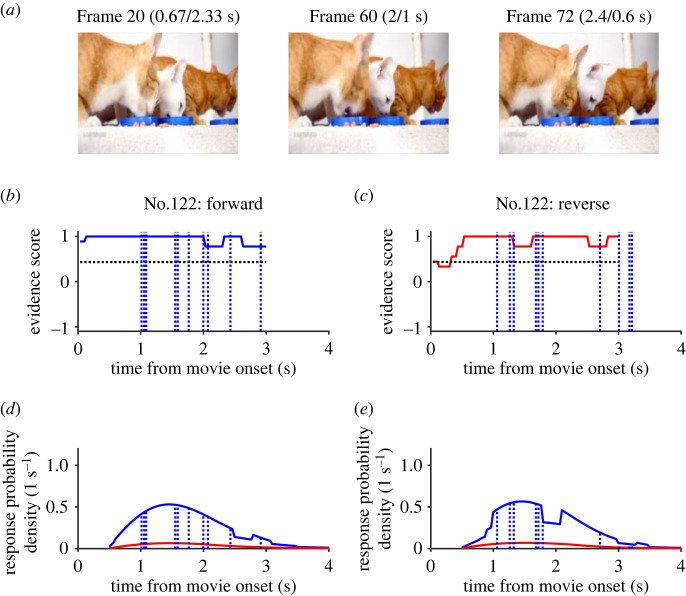
An ambiguous video clip (No. 122). (*a*) Three frames at the same timings as those in figure 3. Note the similarities among the three frames. (*b*,*c*) Responses and evidence scores. Note that the evidence scores (thick lines) remained above the neutral bias (horizontal dotted line). (*d*,*e*) Response probability density functions per second. Note the dominance of the density function for the forward response, which was similar to the Rayleigh function with a peak at approximately 1.5 s. Actual responses (vertical broken lines) occurred over the Rayleigh-like density distributions.

In one ambiguous video clip (No. 122), three cats were continuously eating cat food from their feeding plate during the entire 3 s (figure 5*a*). The participants judged that the video was played in the forward direction whether the video was played forward or in reverse, that is, p(f|f) = 1 and p(r|r) = 0 (p(f|r) = 1). The RT was distributed widely from 1 to 3.2 s in either condition (figure 5*b,c*). The repetitive motions of the head and mouth appeared natural, even when played in reverse.

**Figure 6.**
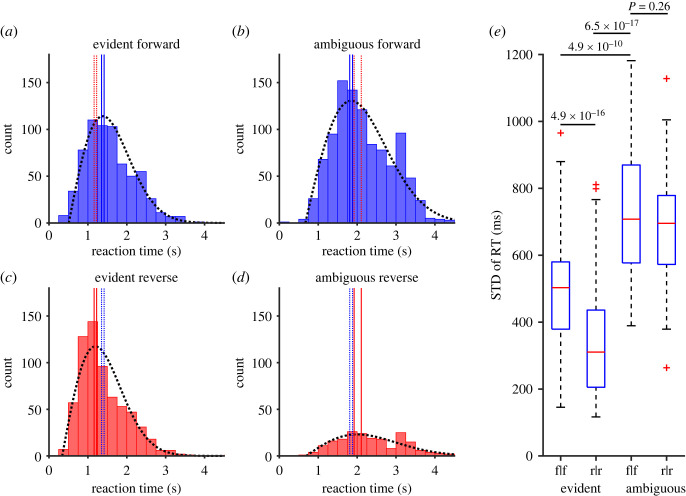
(*a–d*) Distributions of the RT for the forward responses to the forward replay of evident videos (*a*), forward responses to ambiguous videos (*b*), reverse responses to the reverse replay of evident videos (*c*) and reverse responses to ambiguous videos (*d*). Broken curves show the Rayleigh function with a delay fitted to the data. Solid vertical lines show the 95% confidence interval of the peak of each Rayleigh function. Dotted red vertical lines in (*a*,*b*) show the 95% confidence interval of (*c*,*d*) for comparison, respectively. Note that reverse responses were significantly faster in evident videos (*a*,*c*) but slower in ambiguous videos (*b*,*d*). (*e*) Comparison of the standard deviation of the RT among the four groups of trials. Note that the median s.d. was the smallest for the reverse judgement of the evident videos (r|r), less than half of the medians of ambiguous videos (f|f and r|r). *p*-values are shown for four of the six comparisons (Wilcoxon rank sum tests).

These observations, including paradoxically shorter and synchronous reverse responses in the evident video clips, were reflected in the distributions of the RT (figure 6*a–d*), which were well fitted by the Rayleigh distribution (black dotted curves) with small, fixed delays. The goodness of fit by the Rayleigh distribution shows that the hazard rate (probability of response in a unit time) basically increased linearly with time. The peak RT, reflected in the estimated *b* value of the Rayleigh distribution and a delay, was the shortest for the reverse response in the evident reverse replay condition (*b* = 0.85 s, delay = 0.34 s, *b* + delay = 1.19 s, figure 6*c*) with a 95% confidence interval of [1.16 1.23] s (red parallel vertical lines in figure 6*c*). The peak RT increased in the order of 1.38 s (Evident forward responses, figure 6*a*), c.i. = [1.35 1.41] s), 1.85 s (ambiguous forward responses, figure 6*b*, c.i. = [1.81 1.88] s), and 2.01 s (ambiguous reverse responses, figure 6*d*, c.i. = [1.92 2.11] s). These 95% confidence intervals had no overlaps in any of six combinations. Furthermore, the difference in their medians was highly significant in 5 of 6 combinations (*p* < 10^−7^, Wilcoxon rank sum test), with an effect size of 0.59 − 0.83 (AUC), although the difference was still significant in the remaining combination (ambiguous forward responses versus ambiguous reverse responses, *p* = 0.0016, AUC = 0.57).

We further compared the standard deviation of the correct responses (f|f and r|r) across the evident and ambiguous video clips (figure 6*e*). As expected from the example data in figure. 3–5, the median standard deviation was the smallest in the evident reverse responses (median = 310 ms), followed by that in the evident forward responses (503 ms) and the ambiguous reverse (695 ms) and forward (708 ms) responses. The differences were highly significant in five pairs (*p* < 10^−5^, AUC > 0.73), except for the (f|f) and (r|r) responses in the ambiguous trials (*p* = 0.26, AUC = 0.56).

We have shown so far that the reverse responses were faster in the evident video clips in spite of the general bias toward the forward responses. However, this could have resulted from asymmetry in the distribution of evidence: critical events, like the evident splash in video No.1, could have occurred in the later part of the video when it was played in the normal direction. To illustrate the dynamic changes in the strength of evidence, we conducted Experiment 2, in which participants viewed only a 300 ms fraction of the 3 s video clip (figure 1*b*). The evidence score took a value from −1 (all participants judged as reverse) to +1 (all judged as forward).

As expected, the evidence scores for evident clip No. 1 remained positive when it was played forward (blue trace in figure 3*b*) but showed a marked and transient decrease to −1 (red trace in figure 3*c*) when it was played in reverse, just before the ten participants in Experiment 1 made unanimous judgements of reversal. The unnatural convergence of the splash that occurred within a period as short as 300 ms was sufficient for the participants in Experiment 2 to make a unanimous judgement of reversal. As for the evident video clip of the tropical fishes (No. 22), the evidence score reached +1 (or −1) as soon as the replay started in the normal (or reverse) direction (figure 4*b,c*). By contrast, the evidence scores for ambiguous clip No. 122 remained greater than 0.7 most of the time, whether it was played forward or in reverse (blue and red traces in figure 5*b,c*).

**Figure 7.**
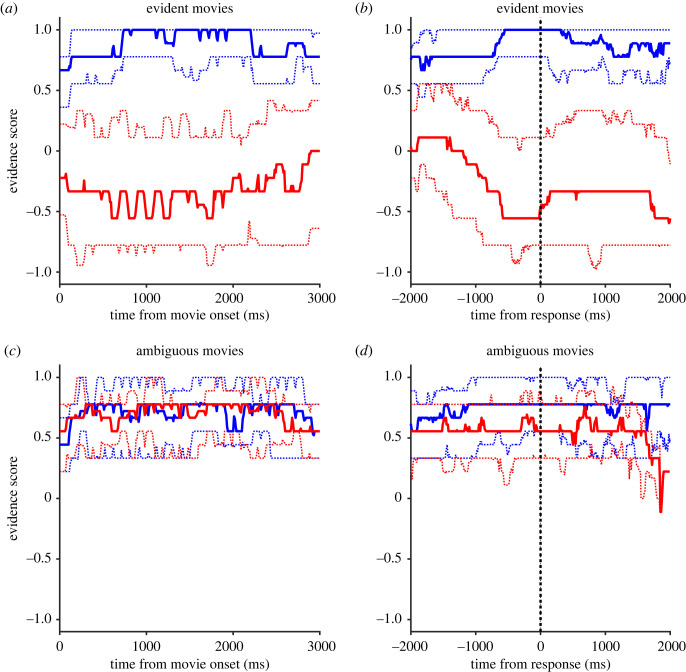
Temporal profiles of evidence scores aligned at movie onset (*a*,*c*) and at response time (*b*,*d*). Solid lines show the median and dotted lines show 25 and 75 percentiles. Colours show the direction of replay (blue: forward, red: reverse). Data are shown for evident (*a*,*b*) and ambiguous movies (*c*,*d*).

When the evidence scores were averaged across the 66 evident video clips, the median evidence scores were almost flat across the replay period whether they were played in the normal or reverse directions (figure 7*a*). However, when the evidence score was averaged around the time of response, it was clear that the median evidence scores increased to +1 (blue) or dropped to −0.5 (red) approximately 0.5 s before making the forward or reverse response (figure 7*b*). As for the ambiguous movies, the median evidence score lingered around +0.7 whether they were played in the normal or reverse direction (figure 7*c*). Separation of the scores before the responses was minimal (figure 7*d*).

By using the evidence score obtained in Experiment 2, we introduced a hazard rate function (*h_f_* for the forward and *h_r_* for the reverse response) whose slope is modified by the evidence score as follows:

3.1 $$h_{f}(t+\mathrm{delay})=t\sqrt{{\left\{k_{f}\;\max(es(t)-c,0)\right\}}^{2}+{\left(1/b_{f}\right)}^{2}}$$

and

3.2 $$h_{r}(t+\mathrm{delay})=t\sqrt{{\left\{k_{r}\min(es(t)-c,0)\right\}}^{2}+{\left(1/b_{r}\right)}^{2}}.$$

Here, *c* denotes a neutral bias of the evidence score (*es*) at which the hazard rate functions simply correlate with time and yield Rayleigh distributions with a peak at *b_f_* and *b_r_*. We fitted the hazard rate model to the data of 120 video clips in Experiment 1 using maximum-likelihood estimation. To ensure the generalizability of our data fitting, we used the leave-one-out technique. The estimated parameters were 0.43 ± 0.032 (mean ± s.d.) for the neutral evidence score (*c*), 0.46 ± 0.000038 s for the delay, and 1.7 ± 0.0046 s and 3.0 ± 0.0013 s for *b_f_* and *b_r_*. The coefficient *k_f_* was 0.043 ± 0.00047, and *k_r_* was 0.040 ± 0.00032.

When ambiguous video No. 122 (cats eating) was played, the evidence score remained above neutral bias whether it was played in the forward or reverse direction (figure 5*b,c*). The resulting response probability functions were similar to the Rayleigh function with a peak at approximately 1.4 s and actual responses distributed according to the predicted distribution. The hazard rate function with a strong bias toward forward judgement (neutral bias = 0.43, *b_f_* = 1.7 s) captured the Rayleigh-like distribution of RT for the forward responses in general.

When evident video clip No. 1 was played forward (figure 3*b*), the evidence score varied around the neutral bias (horizontal dotted line); thus, the response probability density function for the forward judgement was almost identical to a Rayleigh function with a peak at approximately 2 s (figure 3*d*, blue trace). The actual responses were distributed widely over the predicted distribution. In marked contrast, when evident movie No. 1 was played in reverse (figure 3*c*), the evidence score dropped sharply to −1, and the difference from the neutral bias was as large as 1.43. This resulted in a sharp increase in the hazard rate function for the reverse response (*h_r_,* equation (3.2)) with a delay of 0.46 s, which resulted in the formation of a sharp peak of response probability for the reverse judgement at approximately 1 s (figure 3*e*).

When video clip No. 28 (tropical fishes swimming) was played, evidence score was stationarily saturated to +1 (forward) or −1 (reverse) in a symmetrical manner. However, the model predicted faster and more synchronous responses for the reverse replay with a sharp peak of probability density at approximately 1 s (figure 4*e*) than for the forward replay with a dull peak similar to those for the ambiguous video (figure 4*d*). Actual responses of ‘reverse’ occurred within the sharp peaks, showing that the hazard rate function model with a modification term could predict the nearly simultaneous judgement of ‘reversal’ of time's arrow in the evident video clips.

**Figure 8.**
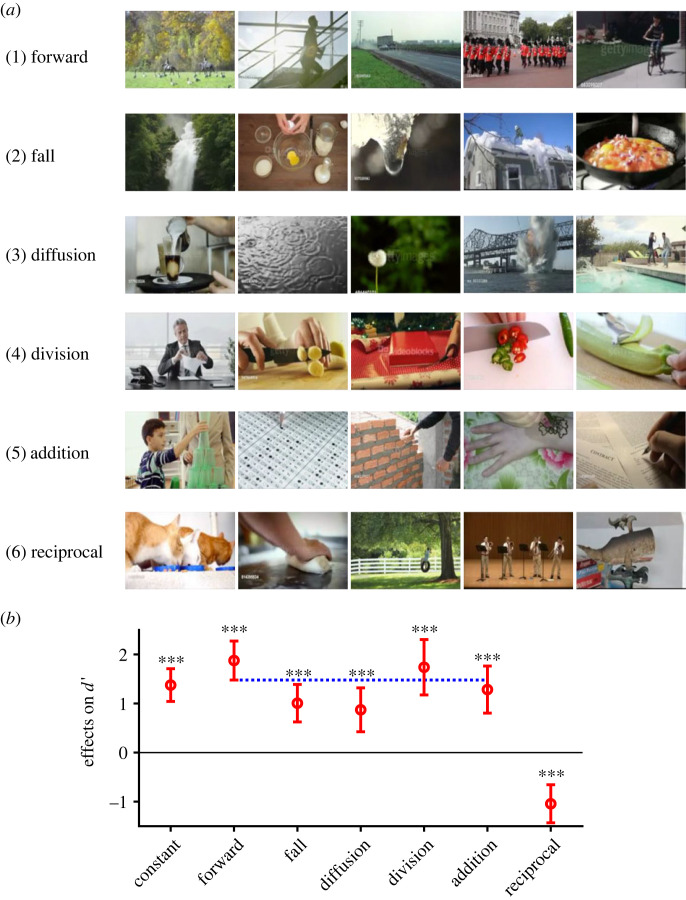
Critical cues sensitive to the reversal of time's arrow. (*a*) Examples for five positive cues (1) forward motion of animals, people and vehicles, (2) free fall of material such as water, food and snow, (3) centrifugal diffusion, spread or explosion, (4) division of material by hand and (5) addition of material or object by hand, in addition to one negative cue (6) reciprocating motion in eating, kneading, swinging and playing musical instruments. (*b*) Estimated coefficients for predicting *d′* from the six cues and a constant. Error bars show the estimated 95% confidence intervals (c.i., 1.96 times s.e.). Asterisks show the level of significance under the null hypothesis that the coefficient was zero (***p < 0.0005; *t*-test). Horizontal dotted line shows the lower bound of the 95% c.i. of the coefficient for the forward motion. Note that the upper bounds of 95% c.i. for the free fall and diffusion did not exceed this line.

However, we still did not know what kind of motion could serve as critical evidence for the reversal of time's arrow strong enough to overturn general bias favouring forward judgement. By observing the evident videos (*n* = 66) one by one, we noted five categories of motion: (1) forward motion of people, animals, or vehicles, (2) free fall, or ballistic motion, under gravitational force, (3) centrifugal diffusion or explosion of small particles, (4) division of some material by hand or a tool and (5) addition, or construction of some material on others by hand. We list examples of videos in each category of motion in figure 8*a*. On the other hand, we noticed that many ambiguous videos contained reciprocating motion, such as head, body and mouth motions while eating (e.g. No. 122) or in conversations, hand and body motions while playing musical instruments, swinging of swings, oscillations of mobiles, etc. (Figure 8*a*, bottom row).

Three experimenters rated the 360 videos as to whether they included each category of motion (1) or not (0). The correlations of the ratings between different raters were 0.67 (r_12_), 0.61 (r_23_) and 0.63 (r_13_) and were highly significant (*p* < 10^−200^). The six scores, averaged across raters, were used to predict the *d′* of each of the 360 video clips. A linear model with the six parameters and a constant was fitted to the data (figure 8*b*). As shown by the estimated coefficients and the 95% confidence intervals (c.i.), all six categories of motion significantly contributed to the prediction of *d’*. Five served as significant cues to help discrimination: (1) forward motion (1.88 ± 0.40, estimated value ± 95% c.i., *p* = 2.2 × 10^−18^), (2) free fall (1.00 ± 0.38, *p* = 4.2 × 10^−7^), (3) diffusion (0.87 ± 0.45, *p* = 0.00016), (4) division (1.74 ± 0.56, *p* = 4.0 × 10^−9^) and (5) addition (1.28 ± 0.48, *p* = 2.8 × 10^−7^). On the other hand, (6) reciprocating motions served as a significant confounding cue (−1.04 ± 0.39, *p* = 2.3 × 10^−7^). It is noteworthy that the contribution from the forward motion was significantly greater than free fall and diffusion but was comparable to division and addition by hand.

## 4. Discussion

In the present study, we used 360 short video clips to test whether individuals are actually able to judge the direction of time's arrow. Participants were able to judge the correct direction in 75% of trials, but there was a clear asymmetry: the correct judgement rate was much greater in the forward replay condition (p(f|f) = 0.91) than in the reverse replay condition (p(r|r) = 0.61). The response bias favouring the forward judgement resulted in faster forward responses in general (figure 2*g*). However, this was not always the case. Participants reported reversal of the arrow faster and more synchronously than they reported normalcy as long as the 66 evident videos with a few critical cues, forward motion, free fall, diffusion, division and addition were played (figures 3 and 4). Linear model analysis further showed that these critical scenes significantly enhanced discrimination between the two judgements (figure 8*b*). Individuals were better able to notice reversal of time's arrow, in situations with the five critical cues in particular, than they were to notice that time is flowing in the normal direction.

### 4.1. Why does the forward response bias predict faster forward responses?

It may be worth clarifying why a response bias favouring one type of response leads to faster responses for the favoured response. Assuming a standard diffusion theory, the bias is expressed as a bias of the starting point (nearer to the threshold of the favoured response) or a bias in the speed of diffusion (greater speed of diffusion toward the favoured response) [17,18]. In either case, the model predicts faster and greater number of responses for the favoured judgement than for the other un-favoured judgement. In agreement with the prediction of the diffusion theory, the forward response was generally faster, in 202 of 294 video clips (68%), after excluding 66 evident videos from the 360 videos. In the evident videos, which consisted of approximately 1/6 of the wide variety of videos, reverse responses somehow overtook the forward response bias.

### 4.2. Forward response bias in the hazard rate model

We did not adopt the diffusion model but adopted a Rayleigh-like hazard function model because the Rayleigh model fit generally better to the distributions of the RT (electronic supplementary material, figure S1). In our model, the response bias favouring the forward judgement was simply captured by a single parameter, *b*, which represented a peak of RT. The parameter was 1.7 s for the forward response (*b_f_* = 1.7 s), nearly half of the parameter for the reverse response (*b_r_* = 3.0 s). The reverse of the parameter represented the slope of the hazard rate function that was nearly twice as large for the forward response (1/1.7 [1/s]) than reverse responses (1/3.0 [1/s]). Additionally, the diffusion model we used for comparison yielded a sigmoid hazard rate function, and the linear hazard rate function with a delay (of the Rayleigh model) captured the gist of the sigmoid hazard rate function yielded by the diffusion model. We thus started from the simple linear hazard rate function with a delay, without assuming any hypothetical diffusion process in the background.

### 4.3. How did the reversal response overtake the other in evident videos?

We can explain how the reversal response overtook the forward response bias in evident video clips using the hazard rate model we developed in the present study (equations (2.3) and (2.4)). When we see a video in which something or somebody moves, we expect that the video is flowing in the normal direction (forward) with a probability of 0.73 (= (1 + *c*)/2, where *c* = 0.47) by default. When the content of the video provides no more or no less evidence than the default baseline, the hazard rate of response increases in proportion to time after a delay of 0.46 s. The slope of the hazard rate function for the forward response (1/*b_f_* = 0.58 [1/s]) is greater than that for the reverse response (1/*b_r_* = 0.33 [1/s]) and yields the Rayleigh distribution with a peak at *b_f_* = 1.7 s for the forward response and another with a peak at *b_r_* = 3.0 s for the reverse response. These ‘baseline’ responses are reflected in the distribution of RT for the ambiguous videos shown in figure 6*b* (forward response) and figure 6*d* (reverse response). Assuming the baseline hazard rate functions, participants respond ‘forward’ in 73% of trials with a peak delay of approximately 2.1 s (= *b_f_* + delay), whereas they respond ‘reverse’ in 27% with a longer peak delay of approximately 3.4 s (= *b_r_* + delay).

However, the evidence score dynamically changes over time from the baseline of 0.43. Notably, the dynamic range for the ‘reverse’ limit of −1, 1.43, is 2.5 times as large as the dynamic range toward the ‘forward’ limit of +1 (0.57). In other words, there is not much room for enhancing the forward judgement due to the baseline bias toward forward, but there is much greater room for enhancing the reverse judgement. The asymmetry in the dynamic range explains why participants made unanimous and synchronous judgements of ‘reverse’ when a critical cue with a strong evidence score of reversal was presented (e.g. splash at frame 72 of video No. 1, figure 3*a*). However, it is worth noting again that such strong evidence is not always available but is provided in some limited occasions, which were categorized into five, in the evident videos.

### 4.4. Generalizability

In our first experiment, we presented natural video stimuli that were 3 s long and occasionally contained critical cues that aided participants in judging the reversal of time's arrow. However, unlike artificial visual stimuli, such as random dot motion stimuli, these cues or evidence for judgement were not uniformly distributed over time and could not be controlled by the experimenter. To overcome this difficulty, we conducted a second experiment in which we assessed dynamic changes in the strength of evidence over 3 s by displaying a fraction of the video (300 ms in duration) to 27 participants. The evidence scores of each video clip displayed a wide range of temporal profiles, with some showing a sharp peak or trough at the beginning of the replay in reverse (e.g. figure 3*c*). However, when the evidence scores were averaged across all the video clips, we found that these cues were distributed almost evenly over the 3 s whether it was played forward or in reverse direction (figure 7*a*). Moreover, when we averaged the evidence score around the time of response, we observed that the median evidence scores increased to +1 (blue) or decreased to −0.5 (red) approximately 0.5 s before making the forward or reverse response, respectively (figure 7*b*). These results indicate that participants responded accurately to the evidence, which could be located at any arbitrary position within the video clips. Based on these findings, we constructed a quantitative model, and we objectively estimated the delay of response from evidence presentation to be 0.46 s.

It is uncertain whether the model developed using evidence scores and responses obtained from 120 video clips can predict responses to novel stimuli. To address this issue, we adopted a leave-one-out procedure, where we excluded a dataset in question from each fit. For example, we take the model predictions for the video clip featuring tropical fishes swimming in the sea (figure 4). The evidence scores were constantly saturated to either end depending on whether it was played in the forward (+1) or reverse (−1) direction (figure 4*b,c*). In spite of the symmetric distributions of evidence, the model predicted faster responses to the reverse replay with a peak at approximately 1 s (figure 4*e*, red curve) compared to the responses to the forward replay (figure 4*d*, blue curve). The asymmetric predictions, which largely depended on the asymmetry in dynamic ranges from the neutral evidence score of 0.43, were verified by the responses of the participants that matched well with the predicted curves of response probability density. It is important to note that the dataset from the video featuring tropical fishes itself was not used for the prediction but just for cross-validation. We expect that the model can predict responses to a range of natural visual stimuli, provided that the temporal profile of the evidence score is available. However, the generalizability of the model needs further verification in the future.

### 4.5. Mechanisms underlying fast reversal detection in critical occasions

Eddington [1] wrote that ‘our reasoning faculty tells us that a reversal of the arrow would render the external world nonsensical’ [1]. This remark was not always true: participants often misjudged that videos were played normally when they were actually played in reverse. However, the remark was true at least when we played evident videos that recorded some critical scenes such as forward motion, free fall, diffusion, and division and addition by hand. Our brain appears to be equipped with a fast-acting ‘reversal’ detector that was designed to work under these critical occasions.

**Figure 9.**
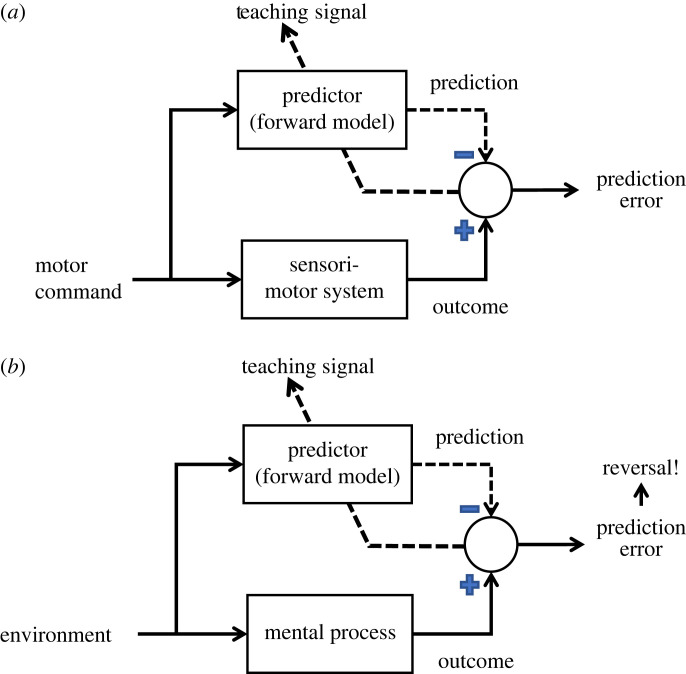
Prediction error as evidence of reversal. (*a*) A predictor, or an internal forward model, of a sensorimotor system. Prediction errors of supervised learning. (*b*) A predictor of any mental process in general, such as mentalizing actions and intentions of others or motions of external objects. The prediction error serves as rapid evidence for reversal, whereas the lack of the prediction error calls for recruitment of a slower cortical process, such as conscious reasoning.

Then, what is the use of the ‘reversal’ detector when time's arrow never reverses in the actual environment? We propose that the ‘reversal’ detector is not designed to detect reversal per se but to yield a prediction error in these critical occasions (figure 9). The concept of a predictor, or an internal forward model, was developed for the predictions of sensorimotor outcomes by the cerebellum [19–21] (figure 9*a*) but was later expanded to the prediction of any mental process in the cerebral cortex, such as mentalizing of others [22,23] (figure 9*b*). With this schema in mind, we notice that all five critical cues for reversal detection can be regarded as prediction errors regarding actions or intentions of others (forward motion of animals, people and vehicles; division and addition by hand) or movements of non-living material (free fall and diffusion). We infer that judgement of normalcy was slower because there was little prediction error, and slower conscious reasoning had to be recruited.

Time's arrow, the subjective ordinary direction of time, is likely to be supported by the acquired predictors and comparators in the brain that are always at work behind the scenes to predict actions or intentions of others. The prediction errors, which are ultimately provided to the cerebellum by way of the inferior olivary nuclei [22,23], are suggested to originate in the cerebral cortices that involve not only the motor cortices but also the parietal cortices [24,25]. In the hierarchical visual cortices, it is proposed that backward projections, from the higher to the lower cortical area, predict and cancel out the sensory input so that only the prediction errors travel from the lower to the higher cortices [26]. The neural bases underlying time's arrow, internal models of the world and prediction error detectors merit further investigation.

## Data Availability

Data are available on Dryad Digital Repository: https://doi.org/10.5061/dryad.cfxpnvx9q [27]. The data are provided in the electronic supplementary material [28].
